# Prevalence and correlation of multiple chemical sensitivity and electromagnetic hypersensitivity with age, sex, and depression in the Japanese population: a retrospective study

**DOI:** 10.1186/s12889-023-16152-2

**Published:** 2023-06-21

**Authors:** Xi Lu, Sachiko Hojo, Atsushi Mizukoshi, Takahiko Katoh

**Affiliations:** 1 Department of Public Health, Faculty of Life Sciences, Kumamoto University, Kumamoto, 860-8556 Japan; 2 Shokei Gakuin University, Natori, Miyagi 981-1295 Japan; 3 Graduate School of Dentistry, Tohoku University, Sendai, Miyagi Japan; 4 Department of Environmental Medicine and Behavioral Science, Kindai University Faculty of Medicine, Osakasayama, Osaka 589-8511 Japan

**Keywords:** Multiple chemical sensitivity, Electromagnetic hypersensitivity, Depression, Sex-based differences, Japanese population

## Abstract

**Background:**

In Japan, there are currently no definitive conclusions regarding the characteristics of multiple chemical sensitivity (MCS) and electromagnetic hypersensitivity (EHS). This study aimed to determine the prevalence and correlation of MCS and EHS with age, sex, and depression in the Japanese population.

**Methods:**

An anonymous self-report questionnaire was distributed to 2,007 participants. Variables such as MCS, EHS, depression score, and demographic characteristics were individually evaluated using the U-test, chi-squared test, and correlation analyses. Moreover, we performed a covariance structure analysis to build a structural equation model.

**Results:**

Older individuals and women were more likely to exhibit MCS and EHS symptoms. Moreover, depression was correlated with MCS and EHS.

**Conclusions:**

Although MCS and EHS are strongly correlated, they exhibit distinct characteristics and symptoms, indicating that they can be regarded as separate conditions.

## Background

Multiple chemical sensitivity (MCS) is characterized by various undefined symptoms in numerous organs, which occur after exposure to extremely small levels of a chemical substance that cannot affect a normal individual [1, 2]. However, several characteristics of MCS remain unclear, including the mechanism underlying its onset, risk factors for onset and aggravation, its relationship with allergic diseases, and psychosomatic and mental disorders. During the last decade, the prevalence of self-reported MCS has been reported to be 3–26%, and it is higher in women than in men [3–8]. Additionally, there are age-related differences in the prevalence of MCS, with adults showing a higher prevalence than older adults and youth [9].

Electromagnetic hypersensitivity (EHS) is characterized by various non-specific symptoms, which differ across individuals. Its symptoms are similar to those of MCS, including headache, fatigue, stress, sleep disturbance, “brain fog,“ short-term memory disturbances, irritability, emotional lability, muscle aches, reduced libido, decreased appetite, skin reactions, and anxiety [10–14]. Although the World Health Organization does not consider EHS as a medical diagnosis and its symptoms do not belong to any known syndrome [15], there have been extensive reports of the symptoms and onset of EHS. Population-based surveys have estimated the prevalence of EHS to be 2.7% in Sweden, 7.2% in Finland [16], 3.2% in California [17], 3.5% in Austria [18], 4% in the UK [11], and 13.3% in Taiwan [19]. Further, 3.0–4.6% of Japanese individuals in the general population may have EHS [13].

EHS and MCS are characterized by non-specific symptoms without a determined toxicological and physiological basis or independent verification [15]. Further, their symptoms largely overlap with those of other functional syndromes. Rea et al. [20] reported that > 80% of patients with EHS present with MCS, which is consistent with the report by Hojo et al. [13]. Belpomme and Irigaray reported that 30% of the patients with MCS have EHS. In addition, EHS and MCS are biologically characterized by low-grade inflammation and an autoimmune response involving autoantibodies against O-myelin [21]. However, elucidation of the causal relationship between chemical substance or EMF exposure and the symptoms has remained challenging; a valid objective test method does not yet exist, and no universal diagnostic criteria have been established.

Moreover, it is important to consider their psychological symptoms. A study on over 2000 patients with self-reported EHS or MCS reported that the symptoms were associated with chronic insomnia, fatigue, depressive tendency, emotional lability, and occasional irritability [21]. In the 1970 and 1980 s, the U.S. government reported that occupational exposure to electromagnetic fields (EMF) led to headaches, sleep disturbances, mood disorders, depression, and memory impairment [22].

Due to insufficient evidence, especially from Japan, there are currently no definitive conclusions regarding the characteristics of MCS and EHS. This study aimed to determine the prevalence and correlation of MCS and EHS with age, sex, and depression in the Japanese population.

## Methods

### Participants

This cross-sectional study was conducted from 2012 to 2015. We included participants aged 18–86 years who resided in 35 prefectures in Japan. Survey requests were made through the communication networks of various organizations to which the co-researchers belonged (academic societies, study groups, universities, vocational schools, architect associations, regional neighborhood associations, and environmental non-profit organizations). We sent a questionnaire and replied to those who agreed to cooperate, asking them to anonymously complete the questionnaire and mail it to the data administrator. We excluded data from patients diagnosed with EHS and MCS by doctors. Valid data described age and sex and showed most Quick Environmental Exposure and Sensitivity Inventory (QEESI) items as completed. The questionnaire was distributed to 2,007 participants; 1,327 returned the questionnaire (response rate, 66.1%). Finally, 1,095 questionnaires contained valid data for analysis.

### Measurements

#### MCS

We used the Japanese version of the Quick Environmental Exposure and Sensitivity Inventory (QEESI©), which was developed by Miller and Prihoda [23, 24] and translated by Ishikawa and Miyata [25], to assess for MCS. The reliability and validity of the Japanese version of QEESI were confirmed by Hojo et al. [26].

The QEESI contains five subscales, each comprising 10 items and 50 subitems. The subscales of the Q4 Masking Index assess whether there is ongoing chemical exposure, with each item being rated by selecting “yes (1)” or “no (0).“ The total score for the Q4 Masking Index ranges from 0 to 10. The other four subscales include Q1 Chemical Intolerance, Q2 Other Intolerance, Q3 Symptom Severity, and Q5 Life Impact, with each item scored from 0 to 10, and the total score ranging from 0 to 100. Hojo et al. [6] provided cut-off values for MCS in the Japanese population (Q1 Chemical Intolerance score ≥ 30, Q3 Symptom Severity score ≥ 13, and Q5 Life Impact score ≥ 17). Accordingly, we used these cut-off values to identify the chemically sensitive population. Moreover, instead of using the aforementioned cut-off values in our structural regression (SR) model, we evaluated MCS using continuous variables of three metrics (chemical intolerance, symptom severity, and life impact).

#### Electromagnetic intolerance

Hojo et al. [13] developed a Japanese translation of the questionnaire originally published by Eltiti et al. [11], modified it to fit the Japanese lifestyle, and confirmed its reliability and validity. The EHS questionnaire comprises the following sections: Demographics, II-1 Symptoms (57 items, q1–q57), and II-2 EMF-producing objects (nine items, q58–q66) considered to cause the symptoms. Next, the participants were asked to rate each item from 0 (not at all) to 4 (quite frequent) for q1–q66, II-3 Reaction to EMFs (q67–q71), and III General health (d1 Well-being, d2 Good health, d3.1 Sleep [fatigue recovery by sleep], d3.2 Sleeping hours per day, and d3.3 Sleep disorder). Subsequently, the participants were asked to rate “yes (1)” or “no (0)” for q69. q68 provides a detailed description of EMF sources and symptoms. For III General health, participants were asked to rate each item for d1, d2, and d3.1 from 0 (not at all) to 4 (quite good) and each item for d3.3 from 0 (not at all) to 4 (quite frequent).

The screening criteria for EHS established by Hojo et al. [13] are II-1 Symptoms total score ≥ 47 points, q67 Sensitive to EMFs ≥ 1 point, and entries of two or more q68 items. We used II-1 Symptoms (57 items, q1–q57) and II-2 EMF-producing objects (9 items, q58–q66) to assess EMF symptoms and EMF-producing objects. Furthermore, we used q67 Sensitive to EMFs and q68 Detailed Description to evaluate the reactions to EMFs. q67 was used to assess the degree of electromagnetic intolerance from 0 (not at all) to 4 (quite frequent), while q68 Detailed Description was used to describe the EMF sources and symptoms. As in the definition MCS, in our SR model, instead of using the cut-off values mentioned above, we evaluated EHS using continuous variables of four metrics (symptoms, producing objects, q67 Sensitive to EMFs, and q68 Detailed Description).

#### Depression

The total health index-depression (IV, 10 items, d5.1–d5.10, added only in the Japanese version of the EHS questionnaire) scale is frequently used in investigations related to industrial hygiene in Japan [27, 28], with the cut-off value for the depressive state being ≥ 22 points [27]. Participants were asked to select the degree of fatigue for each item as follows: “no (1),“ “neither (2),” and “yes (3).” We used this scale to assess participants’ levels of depression.

#### Sociodemographic data

Finally, we collected the demographic characteristics of the participants, including age, sex, education, and employment status.

### Statistical analyses

The parametric and non-parametric data were compared using Student’s t-test and the Mann–Whitney U test, respectively. Further, we performed correlation analyses using variables such as age, sex, depression score, MCS subscale scores (Q1 Chemical Intolerance, Q2 Other Intolerance, Q3 Symptom Severity, Q4 Masking Index, Q5 Life Impact), and EHS subscale scores (total EHS symptom score and total EMF-producing objects, q67 Sensitive to EMFs, and q68 Detailed Description).

Finally, we performed a covariance structure analysis to build an SR model, which is a path model with latent variables, thus combining principles of path and measurement models. This is the most general kind of core model that is widely applied in structural equation modeling (SEM), a collection of procedures that test hypothesized relationships among observed variables [29]. We posited two latent variables (MCS and EHS) and set the following paths: (1) the MCS, EHS, and depression score would be predicted by age and sex; (2) the MCS, EHS, and depression score would be related to each other; (3) MCS would predict Q1 Chemical Intolerance, Q2 Other Intolerance Q3 Symptom Severity, Q4 Masking Index, and Q5 Life Impact; and (4) EHS would predict the total EHS symptom score and total EMF-producing objects, q67 Sensitive to EMFs, and q68 Detailed Description. The model’s fit to the data was examined using a chi-squared test (chi-square mean/degree of freedom [CMIN/DF]). Several studies have described using the comparative fit index (CFI), a revised form of the normed fit index, to compare the fit of a target model to that of an independent or null model. Root mean square error of approximation (RMSEA) can be used to evaluate the fit of data to the model. Additionally, the Akaike information criterion (AIC) is an estimator of out-of-sample prediction error, indicating the relative quality of statistical models for a given data set. Based on conventional criteria, CMIN/DF < 3, CFI > 0.95, RMSEA < 0.08, and a relatively small AIC indicate an acceptable fit; moreover, a good fit is indicated by CMIN/DF < 2, CFI > 0.97, and RMSEA < 0.05 [30]. Statistical analyses were performed using IBM SPSS Statistics version 24.0 and AMOS version 24.0 for Microsoft Windows (IBM, Armonk, NY). All statistical tests were based on two-tailed probability. Statistical significance was set at p < 0.05.

### Ethics approval

This study was approved by the Research Ethics Committees of the Environmental Medical Center, Morioka National Hospital (No. 24 − 01), Sagamihara National Hospital (25-N0.6), Shokei Gakuin University (No. 2020-2), and the Kindai University Faculty of Medicine (No. R02-185). All participants provided informed consent, and the research information was sent to everyone in an anonymized written form. Returning the questionnaire indicated consent to participate in the survey. The contact information of the research representative was also provided on the questionnaire. All study procedures were performed in accordance with the Declaration of Helsinki and its amendments.

## Results

A total of 1327 (66.1%) valid questionnaires were returned. After excluding invalid responses, we included data from 1095 (64.3%) questionnaires in the final statistical analysis. The valid respondents included 323 men (29.5%) and 772 women (70.5%) (mean age [standard deviation]: 39.89 [17.82] years). Compared with men, women were older and had lower education levels (p < 0.001). Moreover, women showed a non-significantly higher prevalence of self-reported MCS and EHS. Table 1 presents the characteristics of the respondents.

**Table 1:** Baseline characteristics of participants

Variables	Total n = 1095 (100%)	Male n = 323 (29.5%)	Female n = 772 (70.5%)	P
***Age*** ^***a***^ **(years)**				
All < 39 40–59 60–86	1095 (39.89 ± 17.82) 559 (24.7 ± 6.49) 347 (49.2 ± 5.66) 189 (67.7 ± 6.13)	323 (38.59 ± 19.39) 184 (23.54 ± 5.68) 77 (49.84 ± 5.77) 62 (69.24 ± 7.03)	772 (40.73 ± 17.11) 375 (25.24 ± 6.79) 270 (49.05 ± 5.63) 127 (66.97 ± 5.51)	**< 0.001** **< 0.001**
***Education*** ^***b***^				
Secondary school High school Junior college /Technical school University Missing	8 (0.7%) 522 (47.7%) 447 (40.8%) 60 (5.5%) 58 (5.3)	0 (0%) 164 (54.8%) 108 (36.1%) 27 (9.0%) 24 (7.4%)	8 (1.1%) 358 (48.5%) 339 (45.9%) 33(4.5%) 34 (4.4%)	**0.001**
***Employment*** ^***b***^				
Unemployed Student Homeworker Part-time worker Full-time worker Missing	75 (6.8%) 363 (33.2%)) 109 (10.0%) 80 (7.3%) 453 (41.4%) 15 (1.4%)	33 (10.4%) 145 (44.9%) 3 (0.9%) 2 (0.6) 134 (41.5%) 6 (1.9%)	42 (5.4%) 218 (28.2%) 106 (13.7%) 78 (10.1%) 319 (41.3%) 9 (1.2%)	**< 0.001**

a: N (Mean ± SD); b: N (%)

Table 2 shows the sex-based differences in the participants’ characteristics and the distribution of the QEESI, EHS, and depression scores. Compared with men, women showed significantly higher scores in all QEESI subscales. Regarding EHS, women showed significantly higher total EHS symptom scores and total EMF-producing objects than men. Similarly, women showed significantly higher scores for q67 Sensitive to EMFs and q68 Detailed Description than men. However, there were no sex-based differences in the depression scores. The prevalence rates of self-reported MCS and EHS were 5.9% and 5.3% in all participants, 4.0% and 3.4% in men, and 6.7% and 6.1% in women. However, there were no significant sex-based differences in the prevalence of MCS (p = 0.08) and EHS (p = 0.07). The prevalence rates of self-reported MCS and EHS were 1.5% in all participants, 0% in men, and 2.1% in women. The sex-based difference was p < 0.01.

**Table 2:** Sex-based differences in participant characteristics and distribution of EHS, MCS, and depression

Variables	Total n = 1095 (100%)	Male n = 323 (29.5%)	Female n = 772 (70.5%)	P
***MCS*** ^***a***^				
Q1 Chemical intolerance Q2 Other intolerance Q3 Symptom severity Q4 Masking index Q5 Life impact	23.88 (21.69) 9.58 (9.79) 14.08(13.39) 3.97 (1.65) 6.45 (11.79)	18.86 (18.78) 7.39 (8.52) 12.06 (11.81) 3.61 (1.63) 5.63 (12.24)	25.98 (22.48) 10.50 (10.14) 14.93 (13.92) 4.13 (1.63) 6.79 (11.58)	**< 0.001** **< 0.001** **< 0.001** **< 0.001** **< 0.001**
***EHS*** ^***b***^				
Total EHS symptom score Total EMF-producing objects score q67 Sensitive to EMFs q68 Detailed description	33.64 (25.53) 3.24 (5.15) 0.28 (0.67) 0.12 (0.33)	29.34 (22.92) 2.77 (4.99) 0.20 (0.59) 0.07 (0.26)	35.44 (26.35) 3.44 (5.18) 0.32 (0.70) 0.14 (0.35)	**0.02** **0.01** **< 0.001** **0.01**
***Depression***	15.81 (5.04)	15.78 (5.21)	15.85 (4.97)	0.95
***MCS Case*** ^***c***^				
- **+**	1030 (94.1%) 65 (5.9%)	310 (96.0%) 13 (4.0%)	720 (93.3%) 52 (6.7%)	0.08
***EHS Case*** ^***c***^				
- **+**	1037 (94.7%) 58 (5.3%)	312 (96.6%) 11 (3.4%)	725 (93.9%) 47 (6.1%)	0.07
***MCS*** **+** ***EHS Case***^**c**^				
- **+**	1079 (98.5%) 16 (1.5%)	323 (100.0%) 0 (0.0%)	756 (97.9%) 16 (2.1%)	**< 0.01**

a: N (Mean ± SD); b. Mean (SD); c: N (%)

MCS, multiple chemical sensitivity; EHS, electromagnetic hypersensitivity; EMF, electromagnetic field

Before establishing an SR model, we estimated the correlations among the QEESI subscale scores, total EHS symptom score, total EMF-producing objects score, q67 Sensitive to EMF, q68 Detailed Description, and depression score. Older age was associated with higher scores for QEESI Q1 Chemical Intolerance, QEESI Q3 Symptom Severity, QEESI Q4 Masking Index, q67 Sensitive to EMFs, and q68 Detailed Description. Contrastingly, younger age was associated with higher QEESI Q5 Life Impact and depression scores. Compared with men, women scored higher for QEESI Q1 Chemical Intolerance, QEESI Q2 Other Intolerance, QEESI Q3 Symptom Severity, QEESI Q4 Masking Index, q67 Sensitive to EMFs, and q68 Detailed Descriptions. All QEESI subscale scores were significantly correlated with the total EHS symptom score and total EMF-producing object score. Moreover, the q67 Sensitive to EMFs and q68 Detailed Description scores were significantly correlated with the scores for most QEESI subscales, except for the QEESI Q4 Masking Index. Depression scores were significantly correlated with the scores for QEESI Q1 Chemical Intolerance, QEESI Q2 Other Intolerance, QEESI Q3 Symptom Severity, QEESI Q5 Life Impact, total EHS symptom score, total EMF-producing objects score, q67 Sensitive to EMF, and q68 Detailed Descriptions (Table 3).

**Table 3:** Correlations between the subscales of the QEESI, EHS, and depression scores with age and sex

	*1*	*2*	*3*	*4*	*5*	*6*	*7*	*8*	*9*	*10*	*11*	*12*
*1. Age*	1	---	---	---	---	---	---	---	---	---	---	---
*2. Sex*	0.05	1	---	---	---	---	---	---	---	---	---	---
*3. QEESI Q1 Chemical Intolerance*	**0.12** ^**^	**0.15** ^**^	1	---	---	---	---	---	---	---	---	---
*4. QEESI Q2 Other Intolerance*	0.05	**0.15** ^**^	**0.59** ^**^	1	---	---	---	---	---	---	---	---
*5. QEESI Q3 Symptom Severity*	**0.08** ^**^	**0.10** ^**^	**0.47** ^**^	**0.55** ^**^	1	---	---	---	---	---	---	---
*6. QEESI Q4 Masking Index*	**0.07** ^*^	**0.14** ^**^	0.02	0.05	**0.15** ^**^	1	---	---	---	---	---	---
*7. QEESI Q5 Life Impact*	**-0.06** ^*^	0.05	**0.32** ^**^	**0.41** ^**^	**0.47** ^**^	-0.01	1	---	---	---	---	---
*8. Total EHS Symptom*	0.05	0.10^**^	**0.35** ^**^	**0.48** ^**^	**0.74** ^**^	**0.16** ^**^	**0.44** ^**^	1	---	---	---	---
*9. Total EMF-producing objects*	-0.02	0.05	**0.28** ^**^	**0.34** ^**^	**0.31** ^**^	**0.06** ^*^	**0.23** ^**^	**0.38** ^**^	1	---	---	---
*10. q67 Sensitive to EMFs*	**0.11** ^**^	**0.08** ^**^	**0.30** ^**^	**0.33** ^**^	**0.30** ^**^	-0.03	**0.19** ^**^	**0.33** ^**^	**0.47** ^**^	1	---	---
*11. q68 Detailed Description*	**0.06** ^*^	**0.09** ^**^	**0.25** ^**^	**0.29** ^**^	**0.23** ^**^	-0.04	**0.15** ^**^	**0.24** ^**^	**0.27** ^**^	**0.54** ^**^	1	---
*12. Depression*	**-0.12** ^**^	0.01	**0.12** ^**^	**0.20** ^**^	**0.40** ^**^	0.03	**0.29** ^**^	**0.45** ^**^	**0.16** ^**^	**0.12** ^**^	**0.09** ^**^	1

EHS, electromagnetic hypersensitivity; EMF, electromagnetic field; QEESI, Quick Environmental Exposure and Sensitivity Inventory

**, p < 0.05, *, p < 0.05

**Fig. 1:**
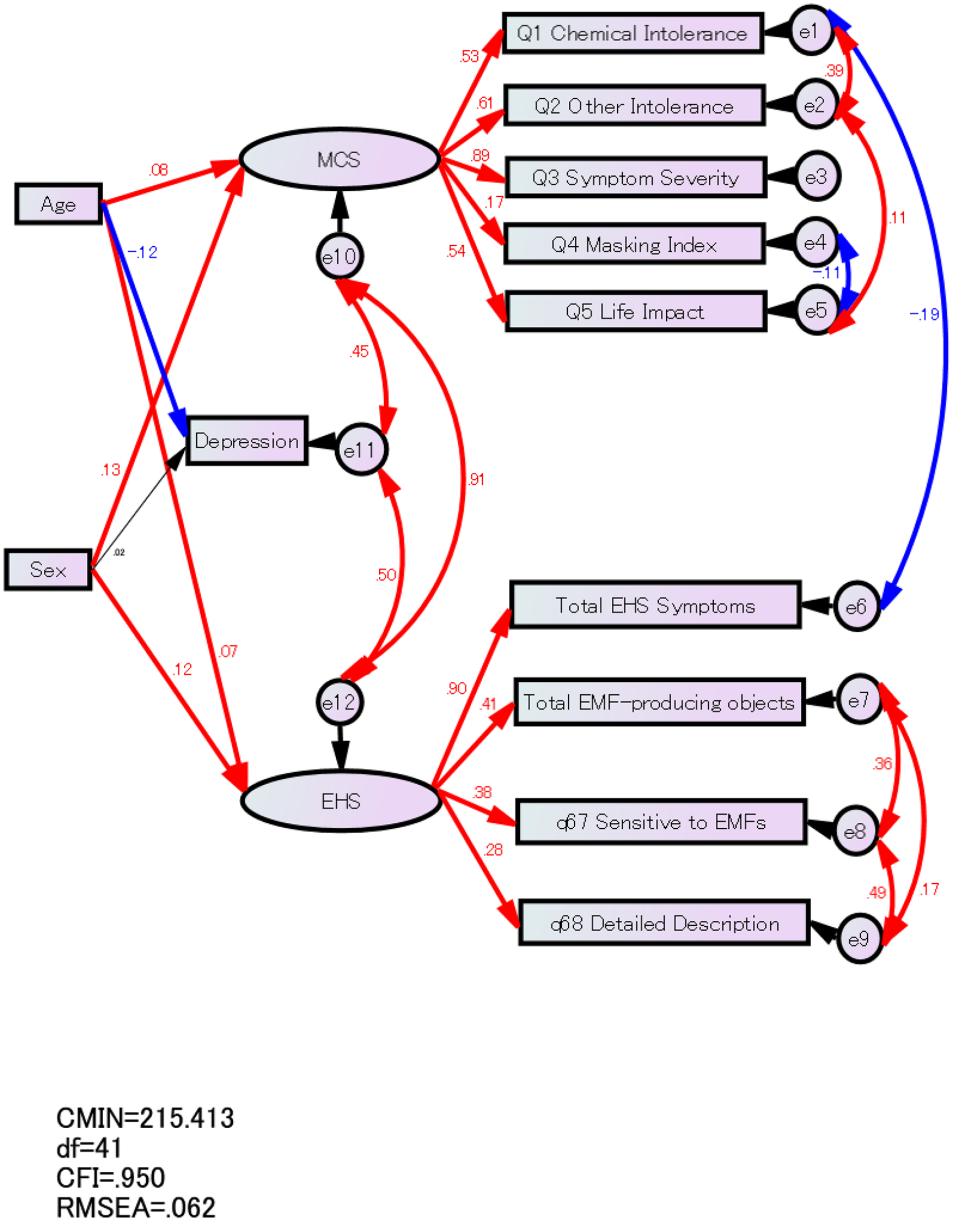
Structural regression model of MCS, EHS, and depression with age and sex MCS, multiple chemical sensitivity; EHS, electromagnetic hypersensitivity; EMF, electromagnetic field; RMSEA, root mean square error of approximation; CMIN, chi-square mean; df, degree of freedom; CFI, comparative fit index

We built an SR model to examine the relationship of MCS with personality and temperament. In the SR model, we set paths between MCS and EHS, depression and MCS, and depression and EHS. Furthermore, to test for sex- and age-based differences, we included factors related to sex and age (Fig. 1). The model showed a good fit with the data: CMIN/DF = 5.98, CFI = 0.95, RMSEA = 0.06 (90% confidence interval: 0.07–0.23). Figure 1 shows the standardized regression coefficients of the variables obtained using the final SR model. The red and blue lines show positive and negative correlation and prediction paths, respectively. In contrast, the paths without statistical significance (p > 0.05) are shown in black letters and thin lines. Older age predicted high MCS, high EHS, and low depression scores (β = 0.08, 0.07, and 0.12, respectively). Further, the female sex predicted high MCS (β = 0.13) and EHS (β = 0.12); however, it was not a significant predictor of depression (β = 0.02). Additionally, MCS was significantly correlated with depression (β = 0.45) and EHS (β = 0.91), while EHS was significantly correlated with depression (β = 0.50).

Regarding the MCS subscales, QEESI Q2 Other Intolerance was positively correlated with QEESI Q1 Chemical Intolerance (β = 0.39) and QEESI Q5 Life Impact (β = 0.11). In contrast, QEESI Q4 Masking Index was negatively correlated with QEESI Q5 Life Impact (β = -0.11). Regarding the EHS subscales, total EMF-producing objects were positively correlated with q67 Sensitive to EMF (β = 0. 36) and q68 Detailed Description (β = 0.17), while q67 Sensitive to EMF was positively correlated with q68 Detailed Description (β = 0.49). Finally, QEESI Q1 Chemical Intolerance demographics were negatively correlated with the total EHS symptoms (β = -0.19).

## Discussion

This study comprehensively explored the relationships among MCS, EHS, and depression in the Japanese population using an SR model and considered the influence of sex and age. The prevalence rates of self-reported MCS and EHS were 5.9% and 5.3%, respectively, consistent with previous Japanese reports [6].

Numerous studies have reported a higher prevalence rate of MCS and chemical intolerance in women than in men [3, 5–8, 31–33], consistent with our findings. Similarly, several studies have reported a higher prevalence of EHS in women than men [16]. However, the lack of sufficiently comprehensive data, especially from the Japanese population, impedes the establishment of clear conclusions. Although we observed no significant sex-based differences in the prevalence of MCS and EHS (p = 0.08; 0.07, respectively), this could be attributed to our small sample size. In our SR model, sex was a predictive factor for MCS and EHS scores. There remains no clear explanation for female predominance. However, several previous studies have offered some explanations, including a tendency for women to experience more health concerns [34] and to be more likely to identify odors than men [35]. According to the 2019 Comprehensive Survey of Living Conditions in Japan, more women than men reported subjective symptoms (especially those aged 20–60 years) [36]. This might be a social explanation for the sex-based differences in the distribution of reported symptoms.

Our results also suggested that age could predict MCS and EHS. Age could be positively correlated with exposure to chemicals and electromagnetic radiation, consistent with recent Swedish and Finnish population-based cohorts that observed a correlation between chemical intolerance with older age [16, 37].

In our SR model, depression was significantly correlated with MCS and EHS. This is consistent with previous reports of a relationship between MCS and mental illness [38]. This could be attributed to the fact that MCS precedes the onset of mental illness [38, 39], indicating that individuals with baseline chemical intolerance showed onset and exacerbation of anxiety within a 5-year period. Another explanation is that MCS is caused by mental illness and that its symptoms represent the somatization of other psychopathologies [40, 41]. MCS is not the only medically unexplainable condition characterized by psychological components. Individuals with chronic fatigue syndrome, heart palpitations, and fibromyalgia frequently exhibit symptoms of anxiety and mood disorders [42–44]. Since this was a cross-sectional study, we could not determine the directionality of the relationship between MCS and mental illness.

MCS showed a significant correlation with EHS. A previous study reported that EHS is correlated with MCS in 30% of the cases and that MCS precedes EHS onset in 37% of these EHS/MCS-related cases. EHS and MCS can be characterized clinically by a similar symptomatic picture and biologically by low-grade inflammation and an autoimmune response involving autoantibodies against O-myelin [21]. Since this was a cross-sectional study, we could not determine the directionality of the relationship between MCS and EHS.

QEESI Q2 Other Intolerances showed positive correlations with QEESI Q1 Chemical Intolerances and QEESI Q5 Life Impact, consistent with previous reports [31, 33, 45]. Moreover, total EMF-producing objects showed positive correlations with q67 Sensitivity to EMF and q68 Detailed Description. Taken together, the extent of exposure was positively correlated with the incidence of symptoms, consistent with previous reports [10, 12–14].

We observed a negative correlation of QEESI Q4 Masking with QEESI Q5 Life Impact (β = -0.11). Miller and Prihoda [23, 24], who developed the QEESI, reported that Q1 Chemical Intolerances might be temporarily masked if individuals are constantly exposed to trace chemical substances from smoking and perfume use. In our study, although Q1 Chemical Intolerance did not show a direct negative relationship with Q4 Masking Index, given its positive correlation with Q5 Life Impact, we believe that after adjusting for age and sex, the relationship between Q5 Life Impact and Q4 Masking Index may have reflected the aforementioned masking effect.

Our SR model showed a negative Q1 Chemical Intolerance (MCS) correlation with total EHS symptoms. This indicates that the severity of chemical intolerance is negatively correlated with the symptomatic response to EHS and may suggest that chemical exposure does not worsen the symptoms of electromagnetic. However, we observed no significant correlation between QEESI Q2 Other Intolerance and total EHS symptoms. MCS is characterized by various undefined symptoms in numerous organs, which occur after exposure to extremely small levels of a chemical substance that cannot affect a normal individual [1, 2]. Conversely, Mizukoshi et al. reported that EMF intolerances were significantly higher in the EHS group than in the MCS group, suggesting that EMF intolerances are subjective symptoms specific to individuals with EHS [46]. If MCS and EHS are of the same disease, we would expect QEESI Q1 and Total EHS Symptoms to be positively correlated; however, our results revealed a negative correlation between Q1 and Total EHS Symptoms. Our findings indicate that although MCS and EHS are strongly correlated, they exhibit different characteristics and symptoms. Thus, it is plausible to consider them as separate conditions, consistent with recent reports [21, 46]. However, we acknowledge the need for further investigation in future studies to validate these findings.

### Limitations

This study has some limitations. First, although we used the SR model to evaluate the overall relationship, this was a cross-sectional study. A prospective cohort study is warranted to elucidate the causal relationships. Second, all data were collected through self-report questionnaires, and recall bias or nocebo effects may exist; accordingly, given the lack of more objective assessments, the results may have been overestimated. Third, this study had a small sample size, which limited the statistical power of some of the findings. Future large-scale studies are warranted to confirm our findings. Fourth, the sample was not a randomly selected representation of the general population. However, as described above, the results of classifying 7,245 randomly sampled individuals from a general population into four categories in the study conducted by Azuma et al. published in 2014 [47] were almost in agreement with the classification results of the controls in this study, suggesting that the latter could be presumed to be representative of a general population. Fifth, our scale information does not include information on the electromagnetic wave exposure of the participants’ residential areas, and thus we could not discuss this part of the effect.

## Conclusions

Our findings suggest that older individuals and women are more likely to exhibit MCS and EHS symptoms in the Japanese population. Further, mental illness, such as depression, was correlated with MCS and EHS. Although MCS and EHS are strongly correlated, they present different characteristics and symptoms. Our results indicate that MCS and EHS may be recognized as different diseases.

## Data Availability

The datasets generated and/or analysed during the current study are not publicly available due [REASON WHY DATA ARE NOT PUBLIC] but are available from the corresponding author on reasonable request.

## Abbreviations

AIC: Akaike information criterion
CMIN/DF: Chi-square mean/degree of freedom,
CFI: comparative fit index
EMF: Electromagnetic fields
EHS: Electromagnetic hypersensitivity
MCS: Multiple chemical sensitivity
QEESI: Quick Environment Exposure Sensitivity Inventory
RMSEA: Root mean square error of approximation
SR: Structural regression

## References

[1] Bartha L, Baumzweiger W, Buscher DS, Callender T, Dahl KA (1999). Multiple chemical sensitivity: a 1999 consensus. Arch Environ Health.

[2] Nethercott JR, Davidoff LL, Curbow B, Abbey H (1993). Multiple chemical sensitivities syndrome: toward a working case definition. Arch Environ Health.

[3] Alobid I, Nogué S, Izquierdo-Dominguez A, Centellas S, Bernal-Sprekelsen M, Mullol J (2014). Multiple chemical sensitivity worsens quality of life and cognitive and sensorial features of sense of SMell. Eur Arch Otorhinolaryngol.

[4] Heinonen-Guzejev M, Koskenvuo M, Mussalo-Rauhamaa H, Vuorinen HS, Heikkilä K, Kaprio J (2012). Noise sensitivity and multiple chemical sensitivity scales: properties in a population based epidemiological study. Noise Health.

[5] Heo Y, Kim SH, Lee SK, Kim HA (2017). Factors contributing to the self-reported prevalence of multiple chemical sensitivity in public facility workers and the general population of Korea. J UOEH.

[6] Hojo S, Mizukoshi A, Azuma K, Okumura J, Mizuki M, Miyata M (2019). New criteria for multiple chemical sensitivity based on the Quick Environmental exposure and sensitivity inventory developed in response to rapid changes in ongoing chemical exposures among japanese. PLoS ONE.

[7] Jeong I, Kim I, Park HJ, Roh J, Park JW, Lee JH (2014). Allergic diseases and multiple chemical sensitivity in korean adults. Allergy Asthma Immunol Res.

[8] Lu X, Hisada A, Anai A, Nakashita C, Masuda S, Fujiwara Y (2020). Study of the correlation between multiple chemical sensitivity and personality using the quick environmental exposure sensitivity inventory questionnaire and the temperament and character inventory. J Occup Environ Med.

[9] Berg ND, Linneberg A, Dirksen A, Elberling J (2008). Prevalence of self-reported symptoms and consequences related to inhalation of airborne chemicals in a danish general population. Int Arch Occup Environ Health.

[10] Baliatsas C, Van Kamp I, Lebret E, Rubin GJ (2012). Idiopathic environmental intolerance attributed to electromagnetic fields (IEI-EMF): a systematic review of identifying criteria. BMC Public Health.

[11] Eltiti S, Wallace D, Zougkou K, Russo R, Joseph S, Rasor P (2007). Development and evaluation of the electromagnetic hypersensitivity questionnaire. Bioelectromagnetics.

[12] Havas M (2013). Radiation from wireless technology affects the blood, the heart, and the autonomic nervous system. Rev Environ Health.

[13] Hojo S, Tokiya M, Mizuki M, Miyata M, Kanatani KT, Takagi A (2016). Development and evaluation of an electromagnetic hypersensitivity questionnaire for japanese people. Bioelectromagnetics.

[14] McCarty DE, Carrubba S, Chesson AL, Frilot C, Gonzalez-Toledo E, Marino AA (2011). Electromagnetic hypersensitivity: evidence for a novel neurological syndrome. Int J Neurosci.

[15] World Health Organization. Factsheets. Electromagnetic fields and public health; electromagnetic hypersensitivity. ; 2005. http://www.who.int/peh-emf/publications/facts/fs296/en/index. Accessed 30 Nov 2015.

[16] Karvala K, Sainio M, Palmquist E, Nyback MH, Nordin S (2018). Prevalence of various environmental intolerances in a swedish and finnish general population. Environ Res.

[17] Levallois P, Neutra R, Lee G, Hristova L. Study of self-reported hypersensitivity to electromagnetic fields in California. Environ Health Perspect. 2002;110;Suppl 4:619 – 23. 10.1289/ehp.02110s4619 PMC1241215 12194896

[18] Schröttner J, Leitgeb N (2008). Sensitivity to electricity–temporal changes in Austria. BMC Public Health.

[19] Meg Tseng MC, Lin YP, Cheng TJ (2011). Prevalence and psychiatric comorbidity of self-reported electromagnetic field sensitivity in Taiwan: a population-based study. J Formos Med Assoc.

[20] Rea WJ, Pan Y, Fenyves EJ, Sujisawa I, Suyama H, Samadi N (1991). Electromagnetic field sensitivity. J Bioelectr.

[21] Belpomme D, Irigaray P. Electrohypersensitivity as a newly identified and characterized neurologic pathological disorder: how to diagnose, treat, and prevent it. Int J Mol Sci. 2020;21. 10.3390/ijms21061915 PMC7139347 32168876

[22] Dwyer MJ, Leeper DB. A current literature report on the carcinogenic properties of ionizing and nonionizing radiation. DHEW Publ. National Institute for Occupational Safety and Health; 1978. pp. 78–134.

[23] Miller CS, Prihoda TJ (1999). The environmental exposure and sensitivity inventory (EESI): a standardized approach for measuring chemical intolerances for research and clinical applications. Toxicol Ind Health.

[24] Miller CS, Prihoda TJ (1999). A controlled comparison of symptoms and chemical intolerances reported by Gulf War veterans, implant recipients and persons with multiple chemical sensitivity. Toxicol Ind Health.

[25] Ishikawa S, Miyata M (1999). Multiple chemical sensitivity criteria and diagnostic test methods Allergol. Immunol.

[26] Hojo S, Kumano H, Yoshino H, Kakuta K, Ishikawa S (2003). Application of quick environment exposure sensitivity inventory (QEESI) for japanese population: study of reliability and validity of the questionnaire. Toxicol Ind Health.

[27] Kawada T, Suzuki S, Kubota F, Ohnishi N, Satoh K (1999). Content and cross validity of the Todai health index depression scale in relation to the center for epidemiologic studies depression scale and the Zung Self-Rating Depression Scale. J Occup Health.

[28] Takeuchi K, Roberts RE, Suzuki S (1994). Depressive symptoms among japanese and american adolescents. Psychiatry Res.

[29] Schumacker RE, Lomax RE (2004). A beginner’s guide to structural equation modeling.

[30] Akaike H (1974). A new look at the statistical model identification. IEEE Trans Automat Contr.

[31] Hojo S, Ishikawa S, Kumano H, Miyata M, Sakabe K (2008). Clinical characteristics of physician-diagnosed patients with multiple chemical sensitivity in Japan. Int J Hyg Environ Health.

[32] Hojo S, Sakabe K, Ishikawa S, Miyata M, Kumano H (2009). Evaluation of subjective symptoms of japanese patients with multiple chemical sensitivity using QEESI(c). Environ Health Prev Med.

[33] Hojo S, Mizukoshi A, Azuma K, Okumura J, Ishikawa S, Miyata M (2018). Survey on changes in subjective symptoms, onset/trigger factors, allergic diseases, and chemical exposures in the past decade of japanese patients with multiple chemical sensitivity. Int J Hyg Environ Health.

[34] Indregard AM, Ihlebæk CM, Eriksen HR (2013). Modern health worries, subjective health complaints, health care utilization, and sick leave in the norwegian working population. Int J Behav Med.

[35] Dalton P, Doolittle N, Breslin PA (2002). Gender-specific induction of enhanced sensitivity to odors. Nat Neurosci.

[36] Ministry of Health, Labor and Welfare. Comprehensive survey of living conditions. ; 2019. https://www.mhlw.go.jp/toukei/saikin/hw/k-tyosa/k-tyosa19/dl/04.pdf. Accessed 17 Jul 2020.

[37] Cui X, Lu X, Hisada A, Fujiwara Y, Katoh T (2015). The correlation between mental health and multiple chemical sensitivity: a survey study in japanese workers. Environ Health Prev Med.

[38] Johnson D, Colman I (2017). The association between multiple chemical sensitivity and mental illness: evidence from a nationally representative sample of Canadians. J Psychosom Res.

[39] Eek F, Karlson B, Osterberg K, Ostergren PO (2010). Factors associated with prospective development of environmental annoyance. J Psychosom Res.

[40] Brown RJ (2004). Psychological mechanisms of medically unexplained symptoms: an integrative conceptual model. Psychol Bull.

[41] Caccappolo-van Vliet E, Kelly-McNeil K, Natelson B, Kipen H, Fiedler N (2002). Anxiety sensitivity and depression in multiple chemical sensitivities and asthma. J Occup Environ Med.

[42] Gracely RH, Ceko M, Bushnell MC (2012). Fibromyalgia and depression. Pain Res Treat.

[43] Kirmayer LJ, Groleau D, Looper KJ, Dao MD (2004). Explaining medically unexplained symptoms. Can J Psychiatry.

[44] Thieme K, Turk DC, Flor H (2004). Comorbid depression and anxiety in fibromyalgia syndrome: relationship to somatic and psychosocial variables. Psychosom Med.

[45] Hojo S, Yoshino H, Kumano H, Kakuta K, Miyata M, Sakabe K (2005). Use of QEESI© questionnaire for a screening study in Japan. Toxicol Ind Health.

[46] Mizukoshi A, Hojo S, Azuma K, Mizuki M, Miyata M, Ogura H (2023). Comparison of environmental intolerances and symptoms between patients with multiple chemical sensitivity, subjects with self-reported electromagnetic hypersensitivity, patients with bronchial asthma, and the general population. Environ Sci Eur.

[47] Azuma K, Uchiyama I, Katoh T, Ogata H, Arashidani K, Kunugita N. Prevalence and characteristics of chemical intolerance: A Japanese population-based study. Arch. 10.1080/19338244.2014.926855 25137616

